# Co-presentation of Cotard’s Syndrome and Autohemophagia: A Report of a Rare Case

**DOI:** 10.7759/cureus.83825

**Published:** 2025-05-10

**Authors:** Ali Z Ansari, Srihita Patibandla, Zayn I Haque, Sanim Choudhury, Dallas J Petroff, Sahar Hafeez

**Affiliations:** 1 Department of Pathology and Laboratory Medicine, William Carey University College of Osteopathic Medicine, Hattiesburg, USA; 2 Department of Psychiatry, Merit Health Wesley, Hattiesburg, USA; 3 Department of Internal Medicine, Trinity Health Grand Rapids, Grand Rapids, USA; 4 Department of Internal Medicine, University of South Florida, Tampa, USA; 5 Department of Internal Medicine, Idaho College of Osteopathic Medicine, Boise, USA; 6 Department of Ophthalmology, Idaho College of Osteopathic Medicine, Boise, USA

**Keywords:** antipsychotic treatment, autohemophagia, cotard’s syndrome, delusional disorder, depersonalization, derealization, nihilistic delusions, psychosis, self-injurious behavior, severe psychopathology

## Abstract

Cotard’s syndrome is a rare neuropsychiatric condition characterized by nihilistic delusions in which patients believe they are dead, do not exist, or have lost their internal organs or bodily functions. Autohemophagia, the act of consuming one’s own blood, is an exceedingly uncommon behavior often associated with underlying psychiatric pathology, particularly within the spectrum of psychosis or severe personality disturbance. We present the case of a 28-year-old female who presented with profound nihilistic delusions consistent with Cotard’s syndrome, alongside repeated acts of deliberate self-injury followed by ingestion of her own blood. She believed she was already dead, lacked internal organs, and that drinking her own blood was the only way to feel corporeal. Her presentation included marked psychomotor retardation, emotional blunting, and fixed delusional beliefs regarding her own nonexistence. Physical examination revealed numerous healing superficial lacerations, and laboratory studies demonstrated mild anemia. Neuroimaging and electroencephalogram (EEG) were unremarkable. After inpatient psychiatric admission, treatment with risperidone and sertraline, alongside supportive psychotherapy, led to partial remission of her symptoms and cessation of autohemophagic behavior. This case highlights the importance of recognizing unusual and severe psychopathological presentations that may involve high-risk behaviors and complex delusional systems. Clinicians should maintain a high index of suspicion for rare comorbid syndromes in psychotic patients presenting with self-injurious behavior, as early identification and multidisciplinary intervention are essential for effective management and prevention of long-term harm.

## Introduction

Cotard’s syndrome is a rare and severe neuropsychiatric condition characterized by nihilistic delusions, in which individuals come to believe they are dead, do not exist, or have lost their internal organs, blood, or bodily functions [1]. First described by Jules Cotard in 1880 as "le délire de négation," the syndrome represents a profound disturbance of self-perception and is typically associated with major depressive disorder with psychotic features, although it has also been observed in the context of schizophrenia, bipolar disorder, and various neurological conditions including epilepsy, stroke, and traumatic brain injury [2]. Clinically, Cotard’s syndrome may present on a spectrum ranging from less severe self-negation to complete denial of bodily existence. The underlying pathophysiology remains incompletely understood, but neuroimaging studies have implicated dysfunction in frontoparietal circuits and limbic structures involved in self-awareness, emotional processing, and integration of bodily states [3]. Despite its rarity, Cotard’s syndrome is of substantial clinical relevance due to the heightened risk of self-harm, neglect, and suicide, often driven by the patient’s conviction that they no longer require care or have already ceased to exist.

Autohemophagia, defined as the intentional ingestion of one’s own blood, is an exceptionally uncommon form of self-injurious behavior. Though most often described in isolated case reports, it has been observed in association with psychotic disorders, dissociative states, and, more rarely, obsessive-compulsive or paraphilic tendencies [4]. Unlike hematolagnia or clinical vampirism, which may carry symbolic, erotic, or ritualistic meanings, autohemophagia typically lacks an overtly sexual or mythological context and instead reflects a deeply pathological response to psychological distress or distorted beliefs about the body [5]. The behavior may involve direct consumption of blood from self-inflicted wounds or from stored samples obtained through deliberate extraction. Chronic engagement in this act carries medical risks, including anemia, infection, and, in rare cases, gastrointestinal complications [6]. The psychological mechanisms underlying autohemophagia remain poorly defined, in part due to its rarity and frequent co-occurrence with other severe psychopathologies that complicate assessment.

## Case presentation

A 28-year-old Caucasian female was brought to the emergency department by her mother due to a three-week history of insidiously worsening self-injurious behavior and verbal expressions of delusional beliefs. The mother reported that the patient had recently begun engaging in deliberate acts of self-harm, including cutting her limbs with sharp instruments and subsequently ingesting the extracted blood. On multiple occasions, the patient was observed storing the blood in small glass containers and later consuming it in what appeared to be a ritualistic manner. The mother noted that the patient often verbalized that she was no longer alive, referring to herself as a "phantom" or "shell," and insisted that ingesting her own blood was the only way she could "remain tethered to the visible world." The patient had no prior psychiatric diagnoses or documented medical conditions. However, she had a long-standing history of emotional withdrawal, social isolation, and intermittent episodes of low mood dating back to adolescence. Despite these longstanding symptoms, she had never undergone formal psychiatric evaluation or treatment prior to the current presentation. She had dropped out of university five years prior and had since been unemployed, living with her mother and avoiding nearly all social contact. Although there was no known history of suicidal attempts, substance use, or significant trauma, the patient had engaged in superficial self-injury as a teenager, which included scratching and minor burning. Her family psychiatric history was unremarkable.

Upon initial evaluation in the emergency department, the patient presented with marked psychomotor retardation and minimal verbal engagement. She appeared disheveled, with matted hair, poor hygiene, and visibly bloodstained clothing. After a prolonged period of silence, she began speaking in a soft, monotone voice. She described feeling certain that she was already dead and stated, “My body moves, but there is nothing inside. I died long ago. I do not breathe or bleed like the living.” When asked about her ingestion of blood, she explained it was necessary to "feel substance" and maintain some semblance of presence in a world she perceived as no longer real. She denied any pleasure or sexual gratification from the act, emphasizing instead a deeply rooted belief that drinking her own blood was the only way to confirm her corporeality. Further psychiatric assessment revealed pervasive nihilistic delusions. The patient believed that her internal organs had decayed, that her brain had stopped functioning, and that she existed in a post-mortem state. She denied active suicidal ideation or homicidal thoughts but expressed a profound conviction that she could not die again. Her affect was blunted, and her insight into her condition was minimal. Although she did not report auditory hallucinations in the conventional sense, she claimed to “hear the echo of emptiness” within her skull. She exhibited significant derealization and depersonalization, often referring to her surroundings as “a thin veil” and stating that she could pass through walls if she stopped concentrating on “pretending to be solid.”

Physical examination revealed multiple superficial lacerations on the forearms, thighs, and anterior chest, consistent with intentional cutting using a fine blade or needle. Some wounds showed early signs of healing, while others were more recent. The patient appeared pale, and her vital signs were within normal limits. Laboratory evaluation showed mild normocytic anemia with a hemoglobin level of 10.4 g/dL and hematocrit of 31.6%. Comprehensive metabolic panel, liver and thyroid function tests, serum B12, folate, and inflammatory markers were unremarkable. Urinalysis and urine toxicology screening were negative. Serological testing for HIV, hepatitis B and C, and syphilis returned non-reactive results. A summary of the patient’s laboratory investigations is provided in Table 1.

**Table 1:** Laboratory test results demonstrated mild normocytic anemia with otherwise unremarkable metabolic, hepatic, thyroid, and inflammatory profiles. Infectious disease screening and toxicology results were negative. MCV: mean corpuscular volume; WBC: white blood cell; BUN: blood urea nitrogen; AST: aspartate aminotransferase; ALT: alanine aminotransferase; TSH: thyroid-stimulating hormone; CRP: C-reactive protein; ESR: erythrocyte sedimentation rate; HIV Ag/Ab: human immunodeficiency virus antigen/antibody; RPR: rapid plasma reagin; VDRL: venereal disease research laboratory

Test	Patient's value	Reference range
Hemoglobin	10.4 g/dL	12.0-16.0 g/dL
Hematocrit	31.6%	36.0-46.0%
MCV	88 fL	80-100 fL
WBC count	6.4 × 10⁹/L	4.0-11.0 × 10⁹/L
Platelet count	280 × 10⁹/L	150-400 × 10⁹/L
Sodium	139 mmol/L	135-145 mmol/L
Potassium	4.1 mmol/L	3.5-5.1 mmol/L
Chloride	102 mmol/L	98-107 mmol/L
Bicarbonate	24 mmol/L	22-29 mmol/L
BUN	12 mg/dL	7-20 mg/dL
Creatinine	0.8 mg/dL	0.6-1.3 mg/dL
Glucose (random)	89 mg/dL	70-140 mg/dL
AST	21 U/L	10-40 U/L
ALT	18 U/L	7-56 U/L
TSH	2.3 µIU/mL	0.4-4.5 µIU/mL
Vitamin B12	460 pg/mL	200-900 pg/mL
Folate	9.6 ng/mL	>3.0 ng/mL
CRP	<1.0 mg/L	<3.0 mg/L
ESR	10 mm/hr	<20 mm/hr
Urinalysis	Negative	Negative
Urine toxicology	Negative	Negative
HIV Ag/Ab	Non-reactive	Non-reactive
Hepatitis B surface antigen	Non-reactive	Non-reactive
Hepatitis C antibody	Non-reactive	Non-reactive
Syphilis (RPR/VDRL)	Non-reactive	Non-reactive

The patient was admitted to the psychiatric inpatient unit and placed on 1:1 observation due to the risk of further self-injury. During the early course of her hospitalization, magnetic resonance imaging (MRI) of the brain revealed no acute abnormalities, masses, or structural lesions. Electroencephalogram (EEG) did not reveal epileptiform activity. Neurocognitive testing demonstrated intact orientation and memory but significant impairments in abstraction, reality testing, and self-referential cognition. Based on clinical presentation and diagnostic findings, a working diagnosis of psychotic disorder not otherwise specified was made. Given the patient’s fixed nihilistic delusions regarding death and corporeal absence, as well as her behavior consistent with self-blood ingestion, a more specific diagnosis of Cotard’s syndrome with concurrent autohemophagia was established following multidisciplinary team consensus.

Pharmacological treatment was initiated with risperidone 2 mg daily, which was later titrated to 3 mg. Sertraline 50 mg daily was introduced on day 5 of admission. Initial compliance with medication was poor; the patient refused treatment, stating that medications were “irrelevant to a dead person.” However, with continued psychoeducation and support from the nursing staff, she eventually agreed to oral administration. Psychotherapeutic interventions were introduced cautiously, focusing on reality orientation, grounding exercises, and supportive therapy aimed at reducing distress rather than challenging fixed delusions in the early stages.

During the first week of hospitalization, the patient remained socially withdrawn, required encouragement for basic activities of daily living, and continued to verbalize delusions of nonexistence. She made several attempts to access sharp objects and was frequently observed engaging in minor skin picking. Over the following week, she gradually began participating in structured group activities, and her self-injurious behaviors decreased. By the end of the third week, her engagement with staff had improved modestly, and she began to express ambivalence about her beliefs, stating, “Maybe my body is real, but I don’t feel like it’s mine.” She no longer attempted to ingest blood and denied current urges to do so. Upon discharge after 24 days of inpatient treatment, the patient demonstrated partial insight and improved affective responsiveness. Although nihilistic delusions persisted to some degree, their intensity and rigidity had lessened. At the time of discharge, the patient carried a primary diagnosis of Cotard’s syndrome with associated autohemophagic behavior, based on her persistent nihilistic delusions and self-injurious blood ingestion. She agreed to outpatient follow-up with a community mental health team and expressed a cautious willingness to continue medication and psychotherapy. Plans were also made for family therapy sessions to support the mother’s understanding of the patient’s condition and promote a stable home environment.

## Discussion

This case presents a rare and clinically important co-occurrence of Cotard’s syndrome and autohemophagia. Cotard’s syndrome involves nihilistic delusions in which individuals believe they are dead, do not exist, or have lost vital organs. It reflects a severe disruption of the minimal self - the fundamental, pre-reflective awareness of one’s body and existence [7]. In this patient, those delusions were accompanied by the compulsive ingestion of her own blood, which appeared to function paradoxically as a means of reasserting her physical presence. Rather than a purely impulsive or compulsive act, her autohemophagia seemed ritualistic and purposeful, possibly serving as a misguided attempt to reaffirm her bodily reality in the face of her belief in nonexistence. This raises important questions about how certain forms of self-injury - particularly those involving bodily ingestion - might serve a symbolic or compensatory role in restoring a disintegrated sense of self.

From a clinical standpoint, the co-presentation of Cotard’s syndrome and autohemophagia significantly increased this patient’s risk and diagnostic complexity. Cotard’s syndrome is frequently associated with profound self-neglect, often manifesting as food refusal or total self-starvation due to the delusional belief that eating is unnecessary, harmful, or impossible [2]. In this case, the patient’s conviction that she lacked internal organs led her to reject all food and fluids, raising concerns about nutritional compromise. However, while mild normocytic anemia was observed, objective evidence of broader malnutrition - such as electrolyte imbalances, vitamin deficiencies, or weight loss - was not present in laboratory findings or on physical examination. Thus, although her clinical behavior suggested a risk for malnutrition, a definitive diagnosis could not be established. The anemia may have been multifactorial, and its direct correlation with nutritional status remains speculative in the absence of more comprehensive indicators. These complications are consistent with previously reported cases in which patients with Cotard’s syndrome experience severe medical deterioration secondary to their delusions, along with an increased risk of suicide, particularly in those who believe they are already dead or perceive death as the only logical resolution [8]. While her autohemophagia might have served as a form of symbolic nourishment, it did not prevent the physiological consequences of starvation and, in fact, exacerbated her anemia. This illustrates the urgency of early medical stabilization and nutritional support in such patients. It also emphasizes the importance of comprehensive medical and psychiatric assessment when patients present with bizarre or high-risk behaviors. Although her neurological workup was unremarkable, her psychomotor slowing, flat affect, and impaired cognition warranted full investigation to rule out structural or metabolic contributors. Interdisciplinary collaboration - particularly among psychiatry, internal medicine, and neurology - was essential in coordinating care and guiding safe treatment.

The patient showed significant clinical improvement with a combined pharmacologic regimen of risperidone and sertraline. Risperidone, a second-generation antipsychotic with dopaminergic and serotonergic antagonism, was selected for its efficacy in reducing psychotic symptoms and its relatively favorable side effect profile. Sertraline, a selective serotonin reuptake inhibitor (SSRI), was initiated in parallel to address the patient's underlying depressive symptoms, which were characterized by anergia, affective flattening, and pervasive hopelessness. The combination of an antipsychotic and antidepressant is supported by case literature and clinical experience, especially in patients whose nihilistic delusions are embedded within a broader depressive or psychotic mood disorder spectrum [9,10]. In this patient, the clinical presentation raised differential diagnostic considerations including major depressive disorder with psychotic features and schizophrenia. However, the absence of prominent mood congruent features, disorganized thinking, or sustained negative symptoms made a primary diagnosis of Cotard’s syndrome with associated autohemophagic behavior more appropriate, though ongoing outpatient evaluation was recommended to monitor for evolving symptomatology. As her depressive and psychotic symptoms began to remit, her autohemophagic behavior also diminished and ultimately ceased, further supporting the interpretation that this self-injurious act was a behavioral manifestation of her distorted self-perception rather than a primary compulsion or impulse-control disorder.

Electroconvulsive therapy (ECT), although not required in this case, remains a first-line consideration in more severe or refractory presentations of Cotard’s syndrome, particularly those complicated by catatonia, suicidal ideation, or life-threatening refusal to eat [11]. Several published reports have described rapid and robust improvements with ECT in similar clinical scenarios, including cases where pharmacologic treatment was ineffective or poorly tolerated [12,13]. In this patient’s case, her relatively early response to combined pharmacotherapy allowed for the deferral of ECT, though it remained an option had her condition failed to improve.

Psychotherapy, while initially limited by poor insight and impaired reality testing, gradually became a more central component of care as her symptoms improved. A supportive, non-confrontational therapeutic approach was used, emphasizing basic trust, safety, and engagement rather than a direct challenge of delusional content. As her insight slowly emerged, therapy shifted toward reinforcing bodily awareness and present-moment grounding techniques, which helped counteract the dissociative and nihilistic elements of her condition. The therapeutic alliance also served as a stabilizing factor, allowing for the exploration of emotional themes and internal conflict without triggering defensive withdrawal or reinforcement of psychotic beliefs. These interventions highlight the importance of a multimodal treatment strategy in addressing both the cognitive and behavioral dimensions of Cotard’s syndrome, particularly when high-risk features such as autohemophagia are present.

## Conclusions

This case highlights a rare co-presentation of Cotard’s syndrome and autohemophagia, revealing how severe disruptions in self-perception can lead to dangerous and unusual self-injurious behavior. The patient’s belief that she was physically nonexistent drove both her refusal to eat and the ingestion of her own blood, emphasizing the clinical risks associated with untreated nihilistic delusions. With timely medical stabilization, antipsychotic and antidepressant therapy, and supportive psychotherapy, the patient made a meaningful recovery. This case demonstrates the need for early recognition, close medical monitoring, and a multidisciplinary treatment approach when patients present with bizarre or high-risk behaviors. Documenting and sharing such cases can improve awareness and guide clinicians in managing similarly complex psychiatric presentations.
